# Eye Movements in Risky Choice

**DOI:** 10.1002/bdm.1854

**Published:** 2015-01-26

**Authors:** Neil Stewart, Frouke Hermens, William J. Matthews

**Affiliations:** ^1^ University of WarwickUK; ^2^ University of AberdeenUK; ^3^ University of CambridgeUK

**Keywords:** eye tracking, decision under risk

## Abstract

We asked participants to make simple risky choices while we recorded their eye movements. We built a complete statistical model of the eye movements and found very little systematic variation in eye movements over the time course of a choice or across the different choices. The only exceptions were finding more (of the same) eye movements when choice options were similar, and an emerging gaze bias in which people looked more at the gamble they ultimately chose. These findings are inconsistent with prospect theory, the priority heuristic, or decision field theory. However, the eye movements made during a choice have a large relationship with the final choice, and this is mostly independent from the contribution of the actual attribute values in the choice options. That is, eye movements tell us not just about the processing of attribute values but also are independently associated with choice. The pattern is simple—people choose the gamble they look at more often, independently of the actual numbers they see—and this pattern is simpler than predicted by decision field theory, decision by sampling, and the parallel constraint satisfaction model. © 2015 The Authors. *Journal of Behavioral Decision Making* published by John Wiley & Sons Ltd.

Risky decisions are central to our behaviour, from frequent but small decisions that have a large cumulative effect (e.g., whether to smoke the next cigarette) to more rare but similarly significant decisions (e.g., where to invest pension funds or whether to start a new business). Economics has been largely concerned with the application of expected utility theory (Bernoulli, 1954; von Neumann & Morgenstern, 1947) as the normative model of risky decision making. But psychology and behavioural economics have taken the model seriously as a description of risky choice behaviour, and this has led to modifications of expected utility to accommodate systematic departures from normative behaviour. The most notable is prospect theory (Kahneman & Tversky, 1979; Tversky & Kahneman, 1992), but there are many significant models (e.g.,Birnbaum, 2008b; Birnbaum & Chavez, 1997; Edwards, 1962; Loomes & Sugden, 1982; Quiggin, 1993; Savage, 1954). These models all have in common the property that the subjective valuation of a risky option is a product of the subjective evaluation of the risks and outcomes involved. These models are constructed almost entirely from choice data and are considered “as‐if” models—they claim that people's choices are consistent with these expected‐value‐like calculations, without claiming that people are actually making these calculations. That is, the models describe the choices but not the process of choosing. Further, these models agree very closely in their choice predictions (Birnbaum, 2004); using measures beyond choice to develop models is therefore appealing.

In parallel, psychologists have developed models of risky decision making that do make claims about the processes that take place. Some process models emerged from the heuristics literature, in which people are assumed to use rules of thumb or shortcuts that may exploit the statistical structure of the environment to approximate optimal decisions (see Gigerenzer & Gaissmaier, 2011, for a review). For risky choice, the best developed heuristic model is perhaps the priority heuristic (Brandstätter, Gigerenzer, & Hertwig, 2006). In this model, people use an ordered sequence of rules to choose, starting with the minimum gain as an initial criterion, proceeding to the probability of the minimum if there is a tie, and finally to the maximum gain if both previous criteria are tied. However, it has been shown that heuristic choice models have difficulties describing choice data (Birnbaum, 2008a, 2010; Birnbaum & LaCroix, 2008) and are not consistent with process data (Johnson, Schulte‐Mecklenbeck, & Willemsen, 2008; Glöckner & Betsch, 2008a; Fiedler & Glöckner, 2012).

Other process models have roots in the perceptual choice literature and involve the idea of repeatedly sampling properties of competing gambles in a series of micro‐evaluations. These samples are accumulated until the evidence sufficiently favours one gamble over the other. This accumulation process, known as a random walk (in the discrete case) or diffusion process (for the continuous case), is ubiquitous throughout psychological modelling of choice (for recent reviews, see Huang, Sen, & Szidarovszky, 2012; Teodorescu & Usher, 2013), particularly in decision making and perceptual choice (e.g., Krajbich, Armel, & Rangel, 2010; Laming, 1968; Loomes, Navarro‐Martinez, Isoni, & Butler, 2012; Ratcliff & Rouder, 1998; Usher & McClelland, 2001; Vickers, 1970). There is good neurophysiological evidence to support these accumulation models (see Gold & Shadlen, 2007, for a review). We pick two examples that differ, primarily, in what is accumulated. In decision field theory (Busemeyer & Townsend, 1993; Diederich, 1997; Roe, Busemeyer, & Townsend, 2001, and see also Wollschläger & Diederich, 2012), attention fluctuates over attribute dimensions on each step in the accumulation process, with the differences in attribute values on the attended dimension accumulated on each step. In decision by sampling (Noguchi & Stewart, 2014; Stewart, 2009; Stewart, Chater, & Brown, 2006; Stewart, Reimers, & Harris, in press; Stewart & Simpson, 2008), a pair of attribute values is compared on each step in the accumulation process, with the number of favourable comparisons for each alternative accumulated.

The last class of model we consider are the parallel constraint satisfaction models (Glöckner & Betsch, 2008b; Glöckner & Herbold, 2011), examples of semantic networks (Collins & Loftus, 1975). In these networks, nodes for gambles and nodes for outcomes are connected by weights that represent probabilities. Excitation of outcome nodes and inhibition between gamble nodes lead to a dynamic pattern of activation spreading through the network that settles with one gamble node more active than the other.

We shall return to these models throughout the article. Researchers have begun to use eye movement data to constrain models of decision making. There is a strong case for a link between eye movements and cognitive processing (e.g., Liversedge & Findlay, 2000; Rayner, 1998, 2009; Yarbus, 1967). Glaholt and Reingold (2011), Orquin and Mueller Loose (2013), and Russo (2011) all provide comprehensive reviews of eye tracking in the decision making literature, so we will just outline some key relevant issues.

## Eye Tracking and Decision Making

The final fixation made at the point of choice is strongly biassed towards the chosen option (Fiedler & Glöckner, 2012; Glaholt & Reingold, 2009b; Krajbich et al., 2010; Schotter, Berry, McKenzie, & Rayner, 2010; Schotter, Gerety, & Rayner, 2012; Shimojo, Simion, Shimojo, & Scheier, 2003; Shi, Wedel, & Pieters, 2013), showing a strong link between choice and eye movements. A core question is whether eye movements drive or reflect choice. Shimojo et al. (2003, see also Simion & Shimojo, 2006, 2007) have argued for a two‐directional gaze cascade mechanism in which we look at options we like more and we like options more the more we look at them, resulting in a positive feedback loop. In judging which of two faces was the more attractive, they found a gradual increase in the likelihood of fixating the ultimately chosen face over the course of a trial—a gaze bias. To infer the direction of causation, eye movements need to be experimentally manipulated. Shimojo et al. (2003) did this and found that forcing participants to orient towards one face more often than the other created a preference shift for that face (about 10%) but that just viewing one face more than the other (without shifting gaze) did not. Armel, Beaumel, and Rangel (2008) also found a modest (about 10%) increase in the probability of selecting a food when viewing was experimentally manipulated so that the food was looked at three times as often as an alternative, and Glaholt and Reingold (2011) found a weak (5%) bias for selecting art photographs blanked on each dwell after a long (400 milliseconds) instead of a short (200 milliseconds) interval in a gaze contingent display (but see Glaholt & Reingold, 2009a, for a null effect of exposure).

Glaholt and Reingold (2011) and Schotter et al. (2010, 2012) explain the bias to the chosen option differently from Shimojo et al. (2003). Glaholt and Reingold (2009b, 2009a, 2011) found a bias for fixations to the ultimately chosen option to be longer in duration throughout the choice and a developing bias to fixate the ultimately chosen option more often later in the choice for both preference and typicality decisions. They interpreted the fixation duration findings as evidence for early selective encoding of information and the fixation frequency findings as evidence for a later comparison and evaluation process. By comparing like and dislike decisions, Schotter et al. (2010) found that the early developing fixation duration bias is towards stimuli people like but that the later developing fixation frequency bias is towards stimuli people choose (but dislike, in the case of dislike decisions). Schotter et al. (2012) found that manipulating the congruency between whether an image is presented in colour or black and white and whether it is older or newer affects only the initial dwell time and not later fixation frequencies—a further dissociation between these effects.

Intuitively, the gaze bias suggests that people have a tendency to look more at options as a preference for those options develops. However, in simulations of the evidence accumulation process, Mullett and Stewart (2014) found that it is sufficient to assume that evidence for an option accumulates at a higher rate while it is being fixated. There is no need for a positive feedback loop. The gaze bias emerges naturally in any accumulator model where the stopping rule is based on the difference in evidence accumulated because, just before a decision, there must necessarily have been a run of fixations to the chosen option for the evidence difference to build up. Retrospective plotting of fixations locked to the point of decision will thus show a bias even when fixations are in fact random.

A second question is whether eye movements are consistent with models of decision making. Early work focussed on the idea that people were calculating something like expected value, and thus, eye movements should be mostly within a gamble, as probability and amount are integrated (Russo & Dosher, 1983). Only partial evidence was found for this hypothesis. Arieli, Ben‐Ami, and Rubinstein (2011), Fiedler and Glöckner (2012), Glöckner and Herbold (2011), and Su et al. (2013) found that eye movements seem to reflect a compromise between within‐gamble eye movements predicted from an expected‐value framework and between‐gamble eye movements predicted from a trade‐off framework.

Glöckner and Herbold (2011) and Fiedler and Glöckner (2012) have also carefully considered the kinds of eye movements predicted by prospect theory, decision field theory, the priority heuristic, and the parallel constraint satisfaction model. They found that more finely balanced choices take longer and involve more fixations, which is inconsistent with literal process interpretations of prospect theory (or expected utility), where people actually multiply probabilities by amounts. Further, the brief fixation durations found in these studies—typically about 200 milliseconds—are not consistent with the longer fixations seen under effortful processing (Horstmann, Ahlgrimm, & Glöckner, 2009) and suggest some simpler automatic process. Eye movements were not consistent with the order of information use in the priority heuristic. The gaze bias (in which a bias to fixate the ultimately chosen option emerges) was not consistent with decision field theory, which predicts no systematic bias in attention emerging over time, but was consistent with the parallel constraint satisfaction model, in which one option becomes accentuated over time.

Finally, Krajbich et al. (2010) and Krajbich and Rangel (2011) have used eye tracking data to constrain a drift diffusion model of choice (much like the decision field theory described earlier). They asked participants to make decisions between different snack options and used fixation data aggregated over trials to set parameters of a drift diffusion model. By assuming that evidence is accumulated faster when an option is fixated than when it is not, they found evidence that the accumulation model can predict the choice and choice time data.

## Towards Complete Statistical Modelling of Eye Movements and Choice

In the present study, we asked people to make a series of simple risky choices. We used off‐the‐shelf generalized linear models to model the frequency of the different types of eye movement and the link between eye movements and choice. This approach subsumes the carefully targeted, theory‐guided comparisons of specific eye movements that others have used and, we hope, represents an exhaustive exploration of the eye movements. We think it is important to take an exhaustive descriptive approach because theory linking choice models to eye movements is relatively underdeveloped. And, by using off‐the‐shelf models, we think our results can be used outside the context of specific theoretical accounts. Throughout, we use the statistical modelling results to sketch constraints for the development of process models of risky decision making. As we describe later, our most frequent recommendation to improve a model's match to the eye movement data is to simplify the model. This is only one study, and so, of course, our results will need replicating in other labs, in more complicated risky decisions, and with different presentation formats.

In the first wave of modelling, we built a Poisson regression model of the frequency of the different types of eye movements during each choice. The coefficients from the Poisson regression capture directly the frequencies of different types of eye movements (e.g., fixations to probabilities vs amounts, and within‐gamble vs between‐gamble comparisons). We used this model to explore how the pattern of eye movements varies over the course of a single choice and how the pattern of eye movements varies across different choices. To anticipate the results, eye movements during risky choices turn out to be quite simple.

In the second wave of modelling, we use a logistic regression model to predict the choices people make from their eye movements at the level of individual trials. In this way, we explore the link between the eye movements people make during a choice and the choice they ultimately make. Again, the logistic regression approach is more exhaustive than specific theoretical comparisons, and we know of only one other study using a related approach (Glöckner, Fiedler, Hochman, Ayal, & Hilbig, 2012). We find that eye movements predict choice independently of the attribute values—a claim no existing analysis can support. Because choices and eye movements are both observed and neither is experimentally manipulated, we do not make claims about the direction of causality.

## Method

### Participants

Forty‐eight participants from the University of Essex participant pool completed the experiment (18 males; median = 21, range = 18–54; age and gender not recorded for one participant). Five additional participants did not begin the experiment because it was not possible to track their eye movements. Data from one participant were replaced without inspection because he or she appeared to fall asleep. Participants received a flat payment of £3 plus between £0 and £2.50 contingent upon their choices. Participants provided written consent in line with the institutional ethical approval.

### Apparatus

Stimuli were presented on an LCD monitor viewed from approximately 85 cm with a 60 Hz refresh rate and a resolution of 1024 × 768. Eye movements were recorded with an Eyelink 1000 desk‐mounted eye tracker (SR Research, Ontario, Canada), which has a reported average accuracy between .25 and .50° of visual angle and RMS resolution of .01° (www.sr‐research.com). We tracked participants' right eye movements using the combined pupil and corneal reflection setting at a sampling rate of 1000 Hz. A chin rest was used to minimize head movements.

### Stimuli

#### Choices

**Figure 1:**
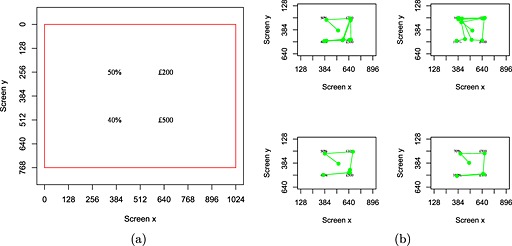
(a) An example screenshot from the experiment. The red border marks the edges of the screen. The axes give coordinates on the 1024 × 768 pixel display. Participants only saw the four attributes (drawn to scale). (b) Fixations in four randomly selected trials from Participant 1. Each green dot represents a fixation. The central dot represents the first fixation to the fixation circle before the attributes appeared. Lines represent saccades and join fixations in order. The last fixation is the fixation at the point when the participant chose

Simple choices were used to keep the number of attributes on the screen to a minimum. Each choice was between two gambles of the form *p* chance of *x* otherwise *1 − p* chance of 0. For example, Figure 1a shows a choice between a 50% chance of £200 (otherwise nothing) and a 40% chance of £500 (otherwise nothing).

Choices were constructed using the following recipe. Probabilities were taken from the set 10%, 20%, 30%, …, 90%, and 100%. Amounts were taken from the set £100, £200, £300, £400, and £500. These simple round numbers make multiplication relatively easy and should promote within‐gamble eye movements (Arieli et al., 2011). Using these attribute values, there are 211 possible choices where one gamble does not stochastically dominate the other. For these choices, we label the lower probability *p* and higher amount *x* and call this gamble the risky gamble. We label the higher probability *q* and the lower amount *y* and call this gamble the safe gamble. Thus, choices were always between a high amount with low probability (the risky *px* gamble) and a low amount with a higher probability (the safe *qy* gamble).

Because we wanted to predict people's choices, we wanted the risky and safe options to be chosen roughly equally often. For this reason, guided by pilot research, we selected only choices where the amount for the risky choice exceeded that of the safe choice by at least £100 (because of risk aversion in our population), leaving 71 choices. The median difference in expected value was £150 with a maximum of £350.

We included an additional four catch choices where one option stochastically dominated the other, to check for participants who were not making sensible choices. Only two participants failed to choose the dominant option on all four occasions, each choosing the dominated option once. We retained all participants in the analysis.

#### Display

The attributes for the two gambles were presented in a square (see Figure 1a). The sides of the square were approximately 100 mm (6.7° of visual angle), measured from the centre of the attributes defining each corner. Thus, when fixating one attribute, the other attributes should fall outside the fovea (2° around fixation) and parafovea (5° around fixation). We chose a font size of 30 points to increase the probability of participants making eye movements between attributes. While we cannot exclude the possibility that participants could read one attribute while fixating another without an experimental test, this is not essential for our design. Our own experience is that it is difficult to read one attribute while fixating another, and this is also what our data suggest, as participants made eye movements on most of the trials. Currency and percentage signs were included on the display so that the type of each attribute could be obviously identified. Attributes were presented in black font on a white background.

### Counterbalancing design

The attributes defining a gamble could be aligned vertically (so that one gamble was on the left and the other was on the right) or horizontally (so that one gamble was above the other). Similarly, the probabilities could appear first (to the left for horizontal alignment or at the top for vertical alignment) or second. To avoid confusion when reading the displays, we counterbalanced these factors between participants in a 2 × 2 design, with 12 participants in each cell. The order of the choices was randomized for each participant. Whether the risky gamble appeared first (at the top for horizontal alignment or on the left for vertical alignment) or second was randomized on each trial. Analysis of the data showed that the counterbalancing and randomization did not affect choices or fixation durations and only affected eye movements in a way readily explained by left–right reading biases.

### Procedure

The lotteries were described to participants in terms of cups of 100 blue and yellow counters, where the proportion of yellow counters determined the probability of winning. The experimenter explained that the probabilities and amounts on the screen should be interpreted as two separate lotteries and that participants should choose the gamble they preferred. Participants were told that they should respond honestly and think about what they would choose if really faced with the two options on the screen. Participants were informed that at the end of the session, one trial would be selected and the lottery chosen on that trial played out for real, giving them the opportunity to acquire a bonus payment proportional to the amount of the chosen lottery. (Specifically, they received 1/200th of the lottery prize; this exchange rate was not revealed until the end of the experiment.)

Before beginning the main part of the experiment, participants undertook practice trials drawn from a different set of choices from those used in the main task. Eye movements were not recorded during this practice phase. Once participants had completed a few trials and indicated that they understood the task, they progressed to the main session. This began with a nine‐point calibration of the eye tracker; calibration was repeated as necessary during the task.

Each trial began with drift correction, during which the participant fixated a small circular target in the centre of the display. The trial was then initiated by a button press from the experimenter that triggered a blank inter‐trial interval with a random duration of 400–800 milliseconds followed by the presentation of the gambles, which remained on‐screen until the participant made a response. When the gambles were vertically aligned, participants pressed “o” or “p” to indicate that they would choose the gamble on the left or right; when the gambles were horizontally aligned, they pressed “p” or “l” to indicate choice of the top or bottom gamble.

## Results

Figure 1b plots the pattern of eye movements on four randomly selected trials from Participant 1. The eye movements are a series of fixations, beginning at the start of the trial on the drift correction target and then dotting between attributes until a choice is made. In our analysis, we describe the pattern of eye movements, explore how the pattern varies across different questions as a function of the probabilities and amounts in the gambles on offer, and finally explore the relationship between the eye movements and the choice ultimately made. An overview of our main findings is presented in Table 1. We note the theoretical importance of the results after each analysis.

**Table 1:**
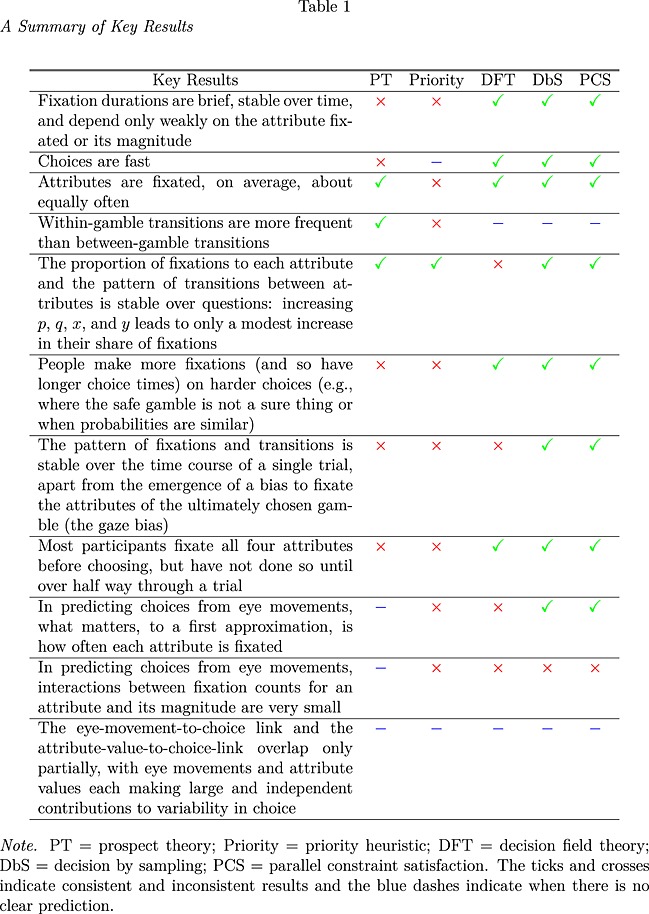


*Note*: PT, prospect theory; Priority, priority heuristic; DFT, decision field theory; DbS, decision by sampling; PCS, parallel constraint satisfaction. The ticks and crosses indicate consistent and inconsistent results, and the blue dashes indicate when there is no clear prediction.

### Fixation clustering

We used a novel procedure for assigning fixations to attributes that contrasts with the standard region‐of‐interest approach. A clustering algorithm was used to assign each fixation as belonging to a particular attribute (or to elsewhere on the screen). Separately for each participant, six Gaussian clusters were fitted in a three‐dimensional space defined by the *x* and *y* locations of a fixation and the fixation duration. Fixation duration was included, as fixations to blank regions of the screen are typically shorter (mean = 212 milliseconds) than to attributes (mean = 275 milliseconds), and so, duration can be used to help classify fixations. The mean vector and covariance matrix for each Gaussian cluster were free parameters and were fitted, together with mixture parameters specifying the proportion of fixations in each cluster, by adjusting parameters to maximize the likelihood of each fixation. The best fit from 100 seed points (where initial cluster parameters were set by randomly allocating each fixation to one of the clusters) was selected for each participant (note that many starting points converged on the same best fit).

**Figure 2:**
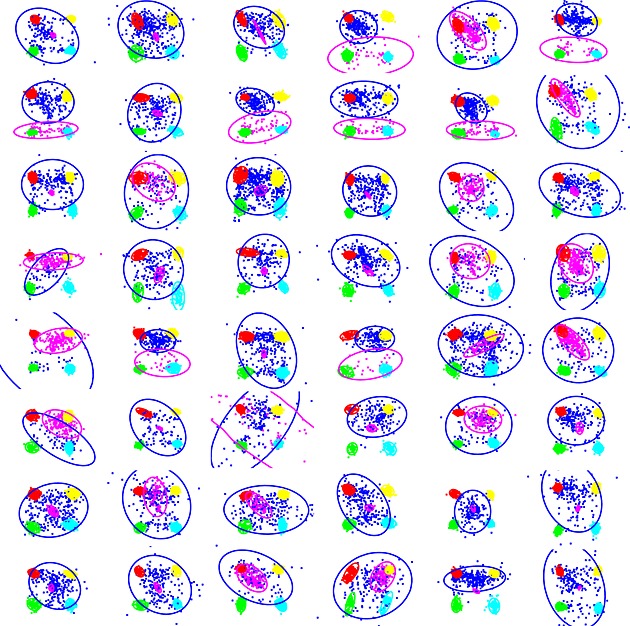
Clustering solutions. Each panel is a separate participant. Each dot is a fixation. The ellipses represent a cluster solution and are equal likelihood contours bounding 95% of the density of the best‐fitting Gaussian clusters. The colour of each dot indicates the cluster that a fixation is most likely to have come from and is thus assigned to

A six cluster solution was chosen, rather than one with more or fewer clusters, as a trade‐off between the simplicity of the solution and the fit to the data. If the number of clusters is selected by comparing the Schwartz's Bayesian information criterion (BIC) (a measure based on the likelihood of the solution corrected for the number of parameters in the model), then a much larger number of clusters are preferred, but this complexity is not required for our purpose of assigning clusters to regions. In addition, a six cluster solution produced very similar results across all participants, identifying a cluster at each attribute in every case. Figure 2 shows the solutions for each participant. The most frequent solution was to identify clusters for each attribute, a cluster for the central fixation, and a diffuse cluster capturing a small proportion of fixations that are obviously to blank portions of the display. A second, less frequent pattern identified a cluster for each attribute but then split the fixations to blank parts of the screen into a higher and a lower cluster. In the following analyses, we assign each fixation to the cluster most likely to have generated it, labelling fixations as to *p*, *q*, *x*, and *y*, or to a blank region (merging the last two categories for each cluster pattern). Ninety per cent of fixations made during a choice were classified as to *p*, *x*, *q*, or *y*.

A second, simpler approach to classifying fixations is to define 100 × 100 pixel regions of interest centred on the four attributes and to classify fixations inside each region as being to their respective attributes, leaving fixations outside these regions unclassified. Fixation classifications from this simple regions‐of‐interest model agreed with classifications from the clustering model for 91.9% of fixations. Of the remaining fixations, 5.3% were unclassified by the regions‐of‐interest method but were assigned to attributes by the clustering method, and 2.9% were unclassified by the clustering method but were assigned to attributes by the regions‐of‐interest method. No fixations were assigned to different attributes by the different methods. In the rest of this paper, we use assignments based on clustering, but the exact method used does not influence the results significantly.

### Choices

The mean proportion of risky picks was close to .50 (mean = .49, median = .48) as we intended. There were large individual differences in the proportion of risky picks, with participant means ranging from .00 to .89 (interquartile range from .36 to .63). We model choices later.

### Fixation duration

Fixation durations are an important focus of analysis in eye movement research. For example, research on eye movements in reading suggests that fixation durations increase with the complexity of the text (Rayner, Pollatsek, Ashby, & Clifton, 2012). Because we have such small targets (e.g., “20%” rather than a whole photograph), we joined consecutive fixations to the same attribute. These were rare and were typically two short duration fixations very close together on an attribute that summed to one normal duration fixation. To preempt the results, we find that fixation durations changed very little over time, across attributes, or with attribute value.

The mean across subjects of the mean fixation duration was 278 milliseconds (SD = 51 milliseconds).

#### Fixation duration over the time course of a trial

**Figure 3:**
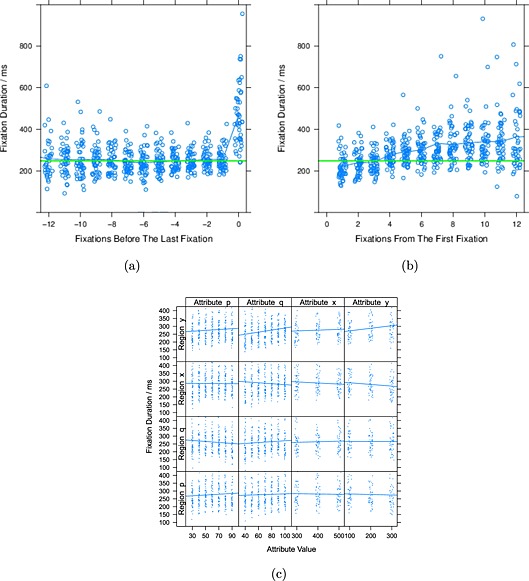
(a) Fixation duration plotted over the time course of a trial. Points are participant means and have been jittered horizontally for visibility. The blue line shows the overall mean at each lag. The horizontal green line shows the overall mean fixation duration (excluding the choice fixation at 0) as a reference. (b) The same data as panel (a), except aligned at the first instead of the last fixation. (c) The effect of attribute values on fixation duration. Each column shows the effect of varying a different attribute value. Each row shows the effect on a different region. Dots are participant means. Lines show the linear effect of attribute value

Figure 3a plots the duration of fixations against trial time. The fixation at the moment of choice (when the participant pressed the button) is on the right, with earlier fixations appearing to the left. The fixation at the moment of choice is longer than the others. Ignoring the final fixation, the mean across subjects of the mean fixation duration was 248 milliseconds (SD = 32 milliseconds). The mean duration across subjects of the final fixation was 467 milliseconds (SD = 149 milliseconds). Mixed effects modelling with a slope for fixation number and by‐subject random effects for the intercept and slope shows that fixations did not get significantly longer over time, *β* = −.63 milliseconds/fixation, MCMC *p*=.47. Consistent with our present result, Janowski and Rangel (2012) found that fixation durations were constant over a trial when choosing between a sure 0 and a lottery offering a gain with some probability otherwise a loss, and Horstmann et al. (2009) found that fixation durations were constant over a trial when choosing which of two cities is the larger given some predictive cues. In contrast, Glöckner and Herbold (2011) found that later fixations are longer. However, they aligned fixations at the first in a choice rather than the last, so their result could reflect increases in the proportion of fixations at a given time being the longer final fixations with increasing fixation number. We see the same pattern as Glöckner and Herbold (2011) when we align our data by the first fixation rather than the last (Figure 3b).

#### Fixation duration and attribute type and value

To examine the influence of the attribute type and value on fixation duration, the mean fixation duration was calculated for fixations to each region (*p*, *q*, *x*, and *y*) for each participant. One might expect longer fixations to amount rather than probability (or vice versa), but these effects are small. Fixation duration does differ over region type, *F*(3, 141) = 2.93, *p*=.039, with fixations to *q* (mean = 264 milliseconds) slightly shorter than fixations to the other attributes (*p* mean = 279 milliseconds, *x* mean = 287 milliseconds, *y* mean = 280 milliseconds).

Each panel in Figure 3c shows the effect of attribute value on fixation duration for fixations to the different attributes. Each column shows the effect of changing a different attribute. Each row shows the effect on fixations to a particular region. For changes to *p*, there is no effect on fixation duration for any region. For changes to *q*, only the duration of fixations to region *y* is affected, *F*(1, 43) = 18.48, *p* <.0001. For changes to *x*, there is no effect. For changes to *y*, only the duration of fixations to regions *x*, *F*(1, 43) = 4.94, *p*=.32, and *y*, *F*(1, 43) = 8.01, *p*=.0071, are affected. (Adjusting the alpha level to control the family‐wise error rate for the 16 slope tests would mean that the effects of *y* were not significant.) Of these effects, the effect of *q* on *y* fixations is largest: As *q* changes from minimum to maximum, the mean fixation duration changes from 243 to 289 milliseconds, an increase of 18%.

#### Discussion

In summary, fixation durations were brief, roughly the same for the different regions *p*, *q*, *x*, and *y*, and did not depend strongly on the values of *p*, *q*, *x*, and *y*. The first entry in Table 1 notes this pattern. Glöckner and Herbold (2011) and Fiedler and Glöckner (2012) also find similarly brief fixations with more complex gambles. Like Glöckner, Fiedler, and Herbold, we suggest that such brief fixations are not consistent with a literal interpretation of expected‐utility‐like models such as prospect theory, in which there is a deliberative multiplication of probabilities and amounts (e.g., in a weighted additive process, Payne, Bettman, & Johnson, 1988, see also Pachur, Hertwig, Gigerenzer & Brandstätter, 2013, for a review of literal interpretations). But brief fixations are more consistent with automatic processes as in the parallel constraint satisfaction model or accumulator models.

Finding that fixation durations are insensitive to attribute value contrasts with the fixation duration effects reported by Glaholt and Reingold (2009b, 2011) and Schotter et al. (2010, 2012) where even the initial fixation is longer for more attractive images. We suspect that the core difference is our use of simple written attribute values compared with the use of photographs in other studies.

Had fixation durations differed over the time course of a trial, this could have been taken as evidence for different processing stages, as Fiedler and Glöckner (2012) also argue. The priority heuristic has an expected‐value screening stage followed by a heuristic stage and so is not consistent with constant duration fixations. But accumulator models and the parallel constraint satisfaction model assume the same cognitive operations over the time course of a decision and so are consistent with constant duration fixations.

### Attribute fixation counts and transitions

In this section, we analyse fixation and transition counts. Because fixation durations were not strongly dependent on the attribute fixated, its magnitude, or its timing within the trial, analysing fixation counts is approximately equivalent to analysing dwell times or their sum, reaction time. Our approach to describing transitions is to build a complete statistical model of the transitions and then to explore how the model changes with the attribute values in each particular choice and over the time course of a trial. By constructing a complete model of the transitions, we can be sure that we have not missed some systematic pattern that might be overlooked by focussing only on certain comparisons.

Table 2 shows the frequency of each kind of transition, aggregated over participants. For example, there were 2547 occasions on which someone was fixating *p* and then made their next fixation to *q*. These totals correspond to a median of 7.5 fixations per choice (ranging from 4 to 11 across participants) or a mean choice time of 2.8 seconds (SD = 1.0 seconds). The zero entries on the negative diagonal are structural: In our analysis, we counted eye movements within the same region as a single fixation (similar to computing dwell times, see Krajbich et al., 2010). Fixations beginning before the onset of the trial or after the response were not included. The totals on the right indicate how often each region was fixated, ignoring the fixation during the button press. The totals at the bottom include the choice fixation but omit the fixation when the display first appeared (which was to the drift correction target to 96.1% of the time)—and thus indicate how often each attribute was fixated during the choice. The lower part of Table 2 gives the same counts as proportions. For example, the very bottom row reports that each of the four attributes was fixated almost exactly one quarter of the time (actual proportions vary from .24 to .26). This means that for the average trial with 7.5 fixations, we would expect an average of just under two fixations to each attribute.

**Table 2:**
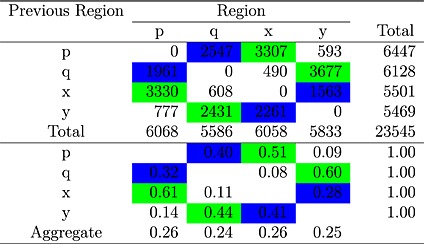
Raw counts of each type of transition summed over participants (top) and the same data as the conditional probabilities of each type of transition (bottom)

*Note*: Green highlighting indicates transitions within gambles. Blue highlighting indicates transitions across gambles within an attribute.

To interpret the transition counts in Table 2, a saturated model of the transition counts was constructed using Poisson regression as described in the Appendix. The first column in Table 3 reports the coefficients from the regression (ignore the later columns for now). These coefficients can be understood as multiplicative adjustments to the transition counts depending on the type of transition. These coefficients completely describe the transition matrix. The (exponentially transformed) *currprob* (for “current fixation is to a probability rather than an amount”) coefficient is .99 and does not differ significantly from 1.00, and indicates that probabilities and amounts were fixated equally often. The *currrisky* (for “current fixation is to the risky gamble rather than the safe gamble”) coefficient is slightly and significantly higher than 1, indicating that people looked a little more at the risky gamble attributes. The *currprob*:*currrisky* interaction coefficient differs significantly from 1, indicating that people viewed *p* and *x* about equally often but viewed *y* slightly more than *q*. That is, there was a small tendency to look more at amounts in the safe gamble. Altogether, the *currprob*, *currrisky*, and *currprob*:*currrisky* coefficients model the frequency of fixations to the different attributes. Although some coefficients are significant, their effects are small and show that, to a first approximation, people fixated each attribute about equally often.

**Table 3:**
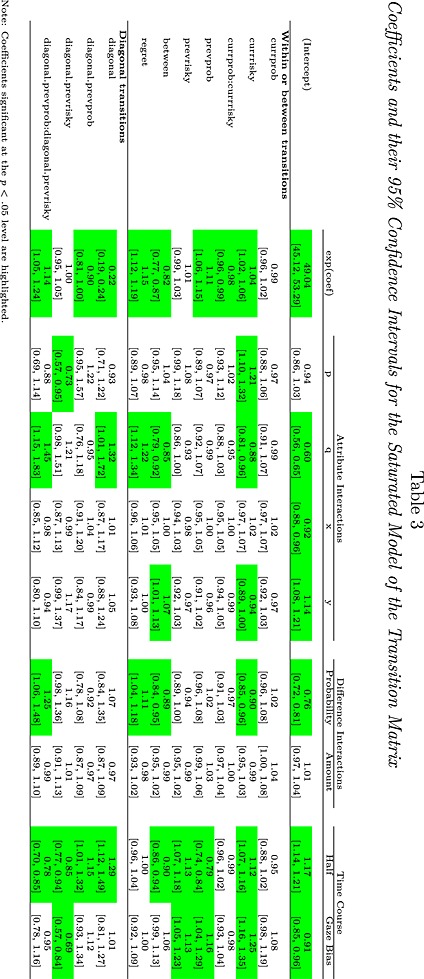
Coefficients and their 95% confidence intervals for the saturated model of the transition matrix

The remaining coefficients *prevprob* through to *regret* model the conditional probabilities of transitions, indicating where transitions are likely to have come from. The *prevprob* coefficient indicates that people were a little more likely to be transitioning from a probability. Given that transitions end on each type of attribute about equally often (*currprob* = .99) and given that the previous attribute of the current fixation is the very same fixation as the current attribute of the previous fixation, this means that people started off more likely to fixate probabilities (we will see this in the next section where we explore how the transition matrix changes over the course of a trial).

There is a large effect of whether the transition is within or between gambles (as the *between* coefficient indicates) with *p* ↔ *x* and *q* ↔ *y* within‐gamble transitions much more likely than *p* ↔ *q* and *x* ↔ *y* between‐gamble transitions. Forty per cent of transitions are between gamble, and 61% of transitions are within gamble. (This split can be directly calculated from Table 2 or derived from the coefficient value of .82: *between*/(*between* + *within*) = (1×.82)/((1×.82) + (1/.82))=.40.) Because we counterbalanced the location and order of the attributes on the screen, the *between* coefficient indicates a true bias for making eye movements within gambles, rather than a bias for horizontal or vertical eye movements.

There is also a smaller interaction (captured by the *regret* coefficient) that indicates that within the overall pattern, people were a bit more likely to make transitions, indicating they thought next about the “downside” rather than the “upside.” For the screenshot in Figure 1a (counterbalanced with probabilities before amounts and gambles read horizontally), this corresponds to a tendency to make clockwise eye movements (e.g., as if people think “50% but only £200 which is less than £400 but I only get this with 40% chance which is less than 50% …”).

#### Discussion

Choices were made quickly in this study—more quickly than in Glöckner and Herbold (2011) and Fiedler and Glöckner (2012), probably because we used simpler gambles with only four rather than eight numbers on the screen. Quick choices are not consistent with literal expected utility accounts where probabilities and amounts are multiplied (Fiedler & Glöckner, 2012), but are consistent with the more automatic processing in accumulator and parallel constraint satisfaction models as noted in Table 1.

The finding that all attributes were fixated about equally often is consistent with all models except the priority heuristic, which predicts that because all of these choices can be made using the “lower probability of the minimum outcome” rule, people will choose before needing to consider the outcomes and so will look at probabilities more often. We note these results in Table 1.

There is considerable variation across studies in the proportion of eye movements that are within gamble rather than between gamble, with between 50% and 80% of transitions within gamble (Arieli et al., 2011; Fiedler & Glöckner, 2012; Glöckner & Herbold, 2011; Rosen & Rosenkoetter, 1976; Russo & Dosher, 1983; Su et al., 2013), probably caused by differences in the number of branches and physical layout. Understanding this variation will require meta‐analysis or further experimental manipulation of factors of interest, but the proportion sits in between that for control tasks that require within‐gamble movements or require between‐gamble movements (Arieli et al., 2011; Su et al., 2013 and also Payne & Braunstein, 1978). Only prospect theory (and other expected‐utility‐like models) predicts that within‐gamble transitions will be more frequent, as we note in Table 1. In its simplest form, decision by sampling assumes that attribute values are sampled at random, which would lead to equally frequent within‐gamble and between‐gamble eye movements. However, as Stewart et al. (2006) suggest, other sampling schemes are possible. The parallel constraint satisfaction model does not make clear predictions about the proportion of within‐gamble versus between‐gamble transitions (Fiedler & Glöckner, 2012). For decision field theory, Fiedler and Glöckner (2012) suggest that the predictions are not clear, while Noguchi and Stewart (2014) argue that because all alternatives are compared at each time point, between‐gamble transitions should dominate. Because the predictions are not clear, Table 1 has dashes for these models.

It is also worth contrasting the general finding that within‐gamble movements are more frequent than between‐gamble movements in risky choice, as described earlier, with the finding from consumer choice that between‐product eye movements are more prominent (Shi et al., 2013).

### Do fixation counts and transition probabilities change as attribute values change?

A question of critical interest is whether the transition matrix varies across the different choice problems. That is, as the values of *p*, *q*, *x*, and *y* vary, does the pattern of fixations that people make vary? Such variation would suggest that the eye movements are driven by the numbers on the screen. The columns under “Attribute Interactions” in Table 3 report the fit of a Poisson regression that includes interactions with the values of *p*, *q*, *x*, and *y*. In the regression, we scaled the values of *p*, *q*, *x*, and *y* so that the interaction coefficients indicate the size of the effect of changing an attribute from its minimum to its maximum.

The “(Intercept)” row in Table 3 tells us how increasing *p*, *q*, *x*, and *y* from minimum to maximum changes the number of fixations in a choice. The value of .94 for *p* indicates that the number of fixations per trial is reduced by 6% (i.e., 1.00–.94) when *p* is increased from minimum to maximum, although this change is not significant. Increasing *q* from minimum to maximum results in 40% fewer fixations. Increasing *x* or decreasing *y* also results in fewer fixations.

The lower entries in Table 3 indicate how the proportions of different kinds of transitions change within the overall total. First, note that despite the large number of eye movements that went into the analysis, most interactions are not significant. The significant interactions with *currrisky* show that as *p* increased, people were 21% more likely to fixate the risky gamble and that as *q* increased, people were 12% less likely to fixate the risky gamble. As *q* increased, people made 15% fewer between‐gamble eye movements and 22% more regret eye movements (as explained in the previous section). As *y* increased, people made 6% fewer fixations to the risky gamble and 7% more between‐gamble eye movements. These effects are all quite modest.

#### Discussion

To summarize the aforementioned results, the transition matrix is quite stable across choices. Changes in *p*, *q*, *x*, and *y* from their minimum to their maximum value had reliable but not large effects on relative frequencies of the different transitions, as noted in Table 1. This finding is consistent with the literal interpretation of prospect theory, as the size of the numbers multiplied is irrelevant. The results are also consistent with decision by sampling and the multi‐attribute version of decision field theory. In these models, attention alternates between probability and amount attributes at each step, with the difference between gambles on that attribute accumulated on each step. However, the result is harder to reconcile with an alternative implementation of decision field theory. This alternative implementation, preferred by Busemeyer (personal communication, 24 January 2011), uses a Savage act‐state representation where differences between outcomes in each state are sampled in proportion to state probabilities. For example, a choice between (i) “a 20% chance of 100 otherwise nothing” and (ii) “a 60% chance of 30 otherwise nothing” would be represented as four states of the world “win A, win B”, “win A, lose B”, “lose A, win B”, and “lose A, lose B” with outcome differences +70, +100, −30, and 0 and probabilities .12, .08, .48, and .32. Each step is an outcome difference, and because states of the world are attended in proportion to their probabilities, the accumulated evidence is, effectively, an expected value. With typical parameters, decision field theory uses hundreds of micro samples to reach threshold. Our eye movement data suggest that this may not be what is happening during choice. The numbers of fixations to *x* and *y* do not increase in proportion with the values of *p* or *q*. That is, if eye movements are indexing attention, people are not attending to states of the world in proportion to their probabilities. Of course, it could be that eye movements are not related to sampling in decision field theory and that it is some intermediate representation constructed after eye movements that is sampled. This could be true, but this effectively makes decision field theory an “as‐if” model with respect to eye movements and leaves their systematic relation to choice unexplained.

While changes in *p*, *q*, *x*, and *y* do not affect the relative share of fixations each attribute receives, they do have strong effects on the overall number of fixations made in a decision. This is the next result in Table 1. This reflects the fact that people choose in fewer fixations (i.e., more quickly—because fixation durations do not vary systematically) when one option is much better than the other. In fact, in the next section, we use an alternative analysis to show that these effects of *p*, *q*, *x*, and *y* occur because people are sensitive only to the difference in probabilities *q* − *p* and because *p*, *q*, *x*, and *y* are correlated with *q* − *p* in the choice set.

Varying decision time across choices is inconsistent with literal expected utility because multiplication time should be largely independent of the magnitudes being multiplied (and if not, there would be the need to explain why the size of the probability multiplicand matters but not the amount multiplicand). But varying decision times are a core property of decision models that posit the accumulation of evidence, such as decision field theory and decision by sampling: Evidence is accumulated for longer in decisions with more finely balanced choices. The priority heuristic also predicts that choice time differs across different decision problems, as rules are applied sequentially until a decision is made. But all of the simple choice problems here are solved at the second rule (“choose the option with the lower probability of the minimum outcome”), so the priority heuristic cannot account for the systematic differences in decision time across choices here.

### Similarity and the transition matrix

Do eye movements depend on the difference in the probability of winning and the difference in the amount to be won? We explored this question by examining whether the transition matrix interacted with the difference between *x* and *y* and the difference between *p* and *q*. The results are shown in the “Difference Interactions” columns of Table 3. We scaled differences to have an overall range of 1, allowing the interaction coefficients to be understood as the effect of differences changing from their minimum to maximum values. The interaction with the intercept indicates that people took 24% fewer fixations to make a decision when the difference *q* − *p* was at its maximum. The bigger the difference in probabilities, the faster people made their decision (Glöckner & Herbold, 2011; Janowski & Rangel, 2012, also find fewer fixations for dissimilar gambles). Our results also show that when the difference in probabilities was larger, people looked less at the risky gamble (i.e., they looked more at the larger probability), made fewer between gamble eye movements, and made more regret direction eye movements, although there is no effect on the proportion of fixations to probabilities. The difference in amounts does not have any significant effect on the transition matrix.

#### Discussion

In interpreting these results, we note that the effects of probability difference and amount difference on the transitions are small in magnitude, with the only large effect being that people chose more quickly when probabilities are more different (Table 1). Fiedler and Glöckner (2012) also explored how the number of fixations varied across choices in a subset of the analysis we completed. They found more fixations to options with higher probabilities, more fixations to options with higher amounts, and more fixations in total when the expected values of gambles were similar. Here, we have replicated this pattern and extended the analysis by exploring how the *relative* frequencies of fixations varied within the overall total. Within the overall total, the effect of attribute values on the proportion of fixations and transitions of each type is modest, consistent with Reutskaja, Nagel, Camerer, and Rangel (2011), who found that the split of fixations between two snacks was not affected by the subjective values of the snacks, and with Janowski and Rangel (2012) who found, in a choice between a sure zero and a lottery offering a gain with some probability otherwise a loss, that the split of fixations between the gain and the loss was independent of the expected value.

### Do fixation counts and transition probabilities change over the time course of a single trial?

**Figure 4:**
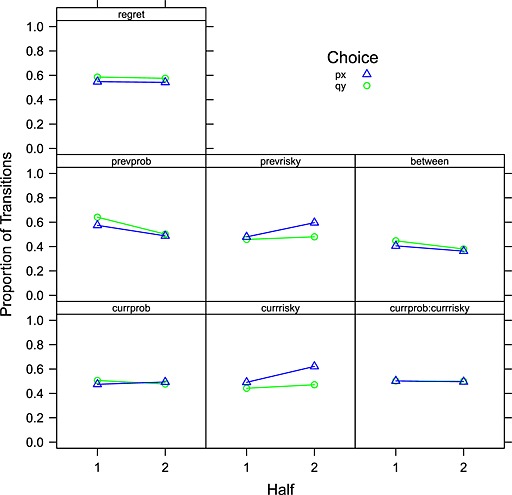
The development of the transition matrix over time, conditional on the final choice. The proportion of transitions of a particular type is plotted for the first and second half of each choice. Trials are split by the final choice. Each panel plots the development of a different type of transition

To explore how the eye movements change as a preference develops, Figure 4 plots how the transition matrix changes over time. Each transition was classified as taking place in the first or second half of a trial. Trials were split into *px* choices and *qy* choices. The final columns in Table 3, under the “Time Course” heading, indicate whether there is a main effect of time (the “Half” column) and whether the effect of time depends on the choice made (the “Gaze Bias” column). The largest effect is a replication of the classic gaze bias shown in the *currrisky* and *prevrisky* panels of Figure 4 in which the probability of fixating the attributes of the ultimately chosen gamble increases over the time course of a trial. The interaction coefficients in the *currrisky* row indicate this, with the 1.12 coefficient indicating a small overall increase in the proportion of fixations to the risky gamble attributes and the 1.25 coefficient indicating that this effect differs depending on the final choice, with the proportion of risky *px* gamble fixations staying about constant over time for safe gamble *qy* choices but increasing for risky *px* gamble choices.

The *prevprob* panel of Figure 4 indicates a drop in the proportion of fixations to probability attributes over time, but the lack of an effect in the *currprob* panel means that this must be due to a bias to fixate probabilities on the first fixation. The *between* panel indicates that the bias towards within‐gamble transitions increases slightly in the second part of a choice (note that Glöckner & Herbold, 2011, find a small effect in the opposite direction).

#### Discussion

This analysis gives us clues about what it is that people do longer in the extra time they take choosing when gambles are similar in probability. The probability of fixating each attribute and the conditional probability of transitioning between attributes are, to a first approximation, stable over time. If eye movements give us a clue to cognitive processing, then their stability indicates that whatever processing is going on, it is the same processing in the first and second halves of a trial (and binning trials more finely into quarters or deciles gives similar results). The same argument can be made from the stability of fixation durations over the time course of a trial, described earlier. The theoretical implication is that there is no evidence for different stages of processing during a choice (this contrasts with consumers' decisions among products, where discrete stages have been identified by Russo & Leclerc, 1994, and Shi et al., 2013). For example, there are no reading phase followed by a multiplying phase as literal expected utility claims, and no reading phase followed by an expected value stage followed by a heuristic stage as the priority heuristic claims. But the constant process over time is consistent with the models like decision field theory, decision by sampling, and parallel constraint satisfaction. This stability over time may not hold for choices between more gambles or between more complicated gambles—but this has not yet been explored in the literature.

The exception to the overall stability in transitions over time is a reliable gaze bias, replicating findings described in the Introduction (Fiedler & Glöckner, 2012; Glaholt & Reingold, 2009b; Krajbich et al., 2010; Schotter et al., 2010, 2012; Shimojo et al., 2003; Simion & Shimojo, 2006, 2007). The gaze bias is a gradually emerging bias to fixate the gamble that is ultimately chosen. Note that our analysis only reveals an association between fixations and choice, and therefore does not permit conclusions about the direction of causality. However, the gaze bias is not predicted by literal expected utility, the priority heuristic, or decision field theory in either the act‐state version (Busemeyer & Townsend, 1993) or the multi‐attribute version (Diederich, 1997; Roe et al., 2001). The priority heuristic and decision field theory are driven by between‐gamble comparisons at all stages and thus necessitate equal attention to each gamble throughout. The gaze bias is also not predicted by the 2N‐ary choice tree model (Wollschläger & Diederich, 2012) because, like the closely related decision field theory, this model employs attribute‐wise comparison of alternatives at each step. (But note that with more than two gambles, the 2N‐ary choice model would predict a gaze bias, as the weakest gamble can by dropped part way through accumulation.) Decision by sampling does predict a gaze bias, because gambles that happen to receive more fixations will more strongly drive the information accumulation process and will therefore be more likely to be chosen, so the retrospective conditioning of fixations on choice will show the effect. Mullett and Stewart (2014) have shown that even a simple accumulation of coin tosses to a fixed head–tails difference produces a plot showing a gaze bias when the proportion of heads at any given time is plotted as a function of the time and the plot is averaged over different length sequences. This means that any model where evidence is accumulated more quickly when an option is fixated than when it is not will produce a gaze bias (Krajbich et al., 2010). Decision by sampling therefore predicts a gaze bias without any top–down link between evidence accumulated so far and fixation probabilities. The parallel constraint satisfaction model also predicts a gaze bias, because processing of subsequent information is influenced by the developing preference as activation settles within the network (Fiedler & Glöckner, 2012).

To summarize the differences in eye movements across choices, people make the same sorts of eye movements for each choice with only small effects of the numbers they are looking at—and they just make more of the same type of eye movements for harder choices.

### The acquisition of information over a trial

**Figure 5:**
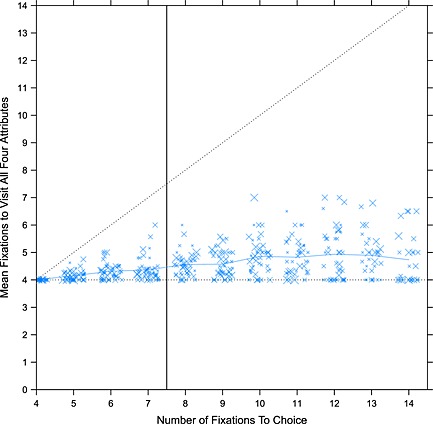
The point during a trial at which all four attributes have been viewed as a function of the total number of fixations. In each column, each point represents a subject mean, with the size indicating the number of observations contributing. The horizontal dotted line marks four fixations and is thus the earliest point at which all four attributes can have been visited. The diagonal dotted line marks the last fixation and thus the last point at which all four attributes can have been visited. The solid blue line is the mean across participants. The vertical line marks the median number of fixations made during a choice

Even under the most efficient strategy, it will require four fixations to read *p*, *q*, *x*, and *y*. In fact, most people read all four attribute values on almost every trial even though, in this experiment, the fourth could sometimes be inferred from the other three. Across participants, the median proportion of trials on which all four attributes are visited is 95%. Figure 5 plots the point during a trial at which all four attributes were visited as a function of the number of fixations during the trial. Most participants lie above the horizontal dotted line, which shows that most participants revisited other attributes before they had visited each attribute at least once. The blue line indicates the mean across subjects, and the vertical black line marks the median total number of fixations until choice. Typically, all four attributes were not visited until just over half way through a choice, and this result is listed in Table 1. It could be that people wait until all four attributes have been visited before beginning the decision process; however, given the similarity of early and late eye movements, people may begin to choose before they have fixated all four attributes.

#### Discussion

With a median of only 7.5 fixations per choice and 4 attributes on the screen, it is somewhat inevitable that people cannot have read each attribute until just over half way through a choice. This means that early on during the trial, people cannot be calculating differences in expected values (or anything similar), as this requires all four attribute values. Earlier, we saw that fixation durations and fixation and transition probabilities are static over the time course of a choice and so provide no evidence that later processing differs from early processing. Thus, if people definitely cannot be calculating expected value in the first half of a choice, they are probably not doing this in the second half of the choice—unless by some coincidence, the eye movements associated with two different processes are the same. Given the literature showing a strong dependence of eye movements on the task performed (e.g., Arieli et al., 2011; Yarbus, 1967), this is unlikely. So, this result, when combined with earlier findings, presents a problem for prospect theory and for the act‐state version of decision field theory, but is consistent with models without a literal multiplication such as multi‐attribute decision field theory, decision by sampling, and the parallel constraint satisfaction model.

### Predicting choices from eye movements

**Figure 6:**
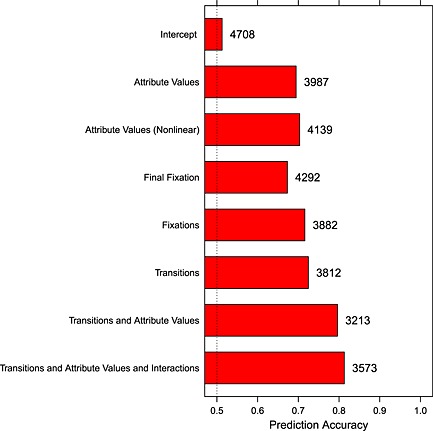
Accuracy with which choices can be predicted based on choice attributes, eye movements, or both. The numbers on the plot give Schwartz's Bayesian information criterion (BIC) for each model, corrected for the nesting of choices within subjects. The BIC values show that better‐fitting models do provide a better account of the data and that the extra model parameters are warranted

If eye movements during choice are related to the decision making process, we would expect to be able to predict choices from eye movements. Figure 6 plots the accuracy in predicting the choice on a particular trial from choice attributes, eye movements, or both (see also Glöckner, Fiedler et al., 2012, for a related approach). Choices were predicted in a logistic regression, where the choice on a trial (risky or safe) was the dependent variable, and the independent variables varied according to the model. Figure 6 also reports Schwarz's BIC, which was used to compare the fits of non‐nested models after differences in the number of parameters are taken into account. We have made the (ultra conservative) correction for the clustering of choices within subjects by replacing the number of observations with the number of subjects, but the pattern is the same whether or not BIC values are corrected. When BIC values are lower for better‐fitting models, this means that the better fits are not due to models with more parameters over‐fitting the data.

In the Intercept Model, where a single free parameter is used to fit the small bias towards making safe picks, the choice predicted as most likely by the logistic regression was the actual choice made on 51.4% of trials.

#### Choices from attribute values

The relationship between choice and attribute values has been explored in 60 or more years of research. Here, we take a very general approach. The Attribute Values Model uses the values of *p*, *q*, *x*, and *y* on each trial to predict the choice on that trial. No eye movement information was used in this model. The model includes all interactions between *p*, *q*, *x*, and *y*, and using one coefficient for each main effect and interaction attains prediction accuracy of 69.5%. Treating *p*, *q*, *x*, and *y* as factors by allowing one coefficient for each level (e.g., allowing one coefficient for the effect of *p* = 20 %, another for *p* = 40 %, and so on…) to allow for nonlinear effects of *p*, *q*, *x*, and *y* and their interactions hardly improved the fit, achieving accuracy of 70.3%, and the corrected BIC values show that the extra complexity of allowing nonlinear value and weighting functions is not warranted. This Attribute Values (Nonlinear) Model is very general and, in its application to these gambles for gains, includes best‐fitting prospect theory with completely free nonlinear functional forms for value and weighting as a special case. That it is not an improvement over the linear model indicates that for these data, nonlinear value and weighting functions are not warranted. The best model, by BIC, is one with linear effects of *p*, *q*, *x*, and *y*, and only the *p* × *q* interaction (in which each probability has a greater effect when the other is small)—with a prediction accuracy of 69.3%. That is, removing the interactions *p* × *x* and *q* × *y*—the very interactions at the core of expected value, expected utility, and prospect theory—reduces the model fit by only .2%, and the increased simplicity of the reduced model more than offsets this small drop in prediction accuracy. In short, the best model contains only additive but independent effects of each attribute, and the *p* × *q* interaction that we saw earlier was important for predicting the number of fixations (or equivalently time) until choice.

#### Choice from eye movements

The Final Fixation, Fixations, and Transitions Models use no information about the values of *p*, *q*, *x*, and *y*. Instead, they use only information about eye movements. Thus, we follow an approach similar to Krajbich et al. (2010), who used a drift diffusion model to predict choices from eye movements (except that we use the eye movements on each trial to predict the choice for that trial rather than using aggregate eye movements to inform model parameters for modelling aggregate choices). Our approach is also similar to that of Glöckner, Heinen, Johnson, and Raab (2012), who used eye movements to predict choice of play after viewing brief video clips of handball games.

The Final Fixation Model uses only the final fixation (i.e., whether the final fixation is to *p*, *x*, *q*, or *y*) to predict choice. Previous work has found that people tend to fixate the option they choose at the point of response (Fiedler & Glöckner, 2012; Glaholt & Reingold, 2009b; Krajbich et al., 2010), and this allows an accuracy of 67.3% in our data.

The Fixations Model uses the number of fixations to each of *p*, *x*, *q*, or *y* to predict choice (ignoring the ordering of fixations). The Transitions Model breaks fixation counts to each attribute down into transitions. That is, the Transitions Model adds information about the ordering of fixations, breaking, for example, the count of fixations to *p* into separate counts for transitions *x* → *p*, *q* → *p*, and *y* → *p*. This extra complexity improves the fit (the BIC value is lower). Table 4 shows the relationship between transitions and choice. Coefficients from the logistic regression were used to model the effect of one more transition of each type on the probability of a risky pick. For example, the value of .20 for the *q* → *p* transition shows that the probability of a risky pick increases from a baseline of .49 to .69 when one more *q* → *p* transition is made. Mixed effects logistic regression modelling with transition counts as fixed effects and full random slopes and intercepts—which thus accounts for the nesting of choices within subjects—and shows that each transition has a significant influence on choice (all *p*s <.002) except for *y* → *q* transitions. Note that the Transitions Model outperforms the Final Fixation Model, too.

**Table 4:** The change in the probability of a risky pick associated with one more transition of each type

Previous region	Region			
	*p*	*q*	*x*	*y*
*p*		–.14	.16	–.28
*q*	.20		.27	–.13
*x*	.18	–.18		–.13
*y*	.24	.00	.27	

The overall pattern in Table 4 is for transitions ending on *p* or *x* to increase the probability of a risky *px* pick and for transitions ending on *q* or *y* to decrease the probability of a risky *px* pick (see columns alternating in sign). We compared the fit with an improper linear model (Dawes, 1979) where the free coefficients for the number of fixations to each attribute are constrained to be equal in magnitude with only their sign retained. This model does only slightly worse on prediction accuracy (2.8% worse), suggesting equal weighting. This pattern is qualified by the tendency for between‐gamble transitions to have an equal or stronger effect than within‐gamble transitions, and it is this secondary trend that gives the Transitions Model the advantage over the Fixations Model. The balancing of the influence of between‐gamble and within‐gamble eye movements was tested by a second improper linear model. Free coefficients for the counts for each type of transition were constrained only to differ for within‐gamble versus between‐gamble transitions. This model does only very slightly worse on prediction accuracy (1.4%). This improper linear modelling is important, because it demonstrates that to a first approximation, transitions are all equal in predicting choice.

Our data contain evidence for both bottom–up influences of eye movements on choice and top–down influences of choice on eye movements. For example, the fact that changes in the value of *x* across questions do not affect the pattern of fixations to *p*, *q*, *x*, and *y* (Table 3, described earlier), but that the number of fixations to *x* does affect the probability of a risky *px* pick (Table 4), indicates that *x* is not affecting how our eyes move, but if we do happen to look more at *x*, we are more likely to choose *px*. In contrast, increases in the value of *p* do lead to more fixations of *p* and *x*, and fixations to *p* and *x* increase the *px* picks. *p* is affecting how we move our eyes and the choices we make. The case of *x* is perhaps best characterised as bottom–up influences of eye movements on choice, but the case of *p* could be characterised as top–down influences of the choice process on eye movements (Shimojo et al., 2003).

#### The interaction between eye movements and attribute values in predicting choices

Does the effect of fixations on choice depend on the value of the attribute being fixated? For example, does looking at *x* increase the probability of a risky pick more when *x* is larger? Larger attribute values are more likely to terminate comparisons and lead to a choice in the priority heuristic. Larger attribute values should lead to bigger steps in the accumulation process in decision field theory and more probable steps in the accumulation process in decision by sampling. Incorporating attribute‐value‐by‐fixation‐count interactions into a mixed effects logistic regression model with fixation count and attribute value fixed effects (and full random effects) significantly improved the fit of the model, *χ* ^2^(8) = 38.7, *p* <.0001. Only the attribute‐value‐by‐fixation‐count interaction for *p* reached significance, *p* <.0001: Fixations to *p* increased the probability of a risky pick more when *p* was large. However, although significant, the interaction effect is small, increasing accuracy by only .3%. The penultimate row of Table 1 notes that most models predict that fixations should count more when the values fixated are large—which is not consistent with the very small and null effects observed.

We have repeated the aforementioned analysis replacing fixation counts for an attribute with the total duration of fixations to that attribute. Using fixation durations instead of counts predicts choice significantly more poorly (a drop of 2.4%). Adding interactions with attribute values improves this fit significantly, but as for fixation counts, the improvement is very small (.1%).

#### Choice from eye movements and attribute values

The last model in Figure 6, the Transitions and Attribute Values Model, shows that by using attribute values and transitions together, an accuracy of 79.6% can be obtained. Obviously, using both attribute values and transitions together is an improvement over either attribute values alone (Attribute Values Model) or transitions alone (the Transitions Model), as shown by the BIC values. But to what extent does this improvement reflect the fact that transitions and attribute values are explaining different variance in the choices? The Nagelkerke *R* ^2^ measure for each model provides this information. Nagelkerke *R* ^2^ = 0 means that no variance in choices is explained; Nagelkerke *R* ^2^ = 1 means that choices would be perfectly predicted. Nagelkerke *R* ^2^, rather than one of the other pseudo *R* ^2^ measures, was chosen because these *R* ^2^ values are additive for independent predictors. Table 5 shows the Nagelkerke *R* ^2^s for the models. If the Attribute Values Model and the Transitions Model were predicting completely independent variance, then the Nagelkerke *R* ^2^ for the combined Transitions and Attribute Values Model would be the sum for the separate models, .59. That means that of the .27 of the variance that the Attributes Model could contribute on top of the Transitions Model, .09 is already explained. And vice versa, of the .32 of the variance that the Transitions Model could contribute on top of the Attributes Model, .09 is already explained. Thus, about one third (i.e., .09/.27 or .09/.32) of the variance accounted for by transitions is also explained by attribute values, and vice versa.

**Table 5:** Nagelkerke *R* ^2^ for choice models

Model	Nagelkerke −*R* ^2^
Attributes	.27
Transitions	.32
Attributes and transitions	.50

#### Discussion

The final findings in Table 1 concern the link between eye movements and choice. Of course, these observations are correlational, and so, we cannot and do not make claims about causality. The findings earlier were as follows: (i) Eye movements predict choice a little bit better than, for example, applying prospect theory to the attribute values. (ii) The relationship between eye movements and choice is reasonably simple: More fixations to *p* and *x* are associated with a higher probability of a risky *px* choice, and more fixations to *q* and *y* are associated with a higher probability of a safe *qy* choice. Weighting fixations to each type of attribute equally hardly reduces the predictive power of this model, so, to a first approximation, all fixations contribute equally. This pattern contains a curious result: Even the rare and uninformative between‐gamble between‐attribute transitions (*x* ↔ *q* or *y* ↔ *p*) predict choice as strongly as the sensible within‐attribute between‐gamble transitions. (iii) A fixation to an attribute is not worth more if the attribute value is larger. Together (ii) and (iii) suggest a somewhat simple accumulation of evidence: If something more complicated were accumulated, then the uninformative diagonal transitions should have no weighting, and fixations involving larger attribute values should have more weighting. We may therefore sketch a very simple first‐order model: *In making mostly sensible within‐attribute between‐gamble or between‐attribute within‐gamble eye movements, people just choose the gamble they look at more*. In this model, it is fixation counts that are accumulated. In decision field theory, each gamble is considered at every time step, so decision field theory does not predict that more fixations to a gamble should increase the likelihood that it is chosen. Decision field theory and the related 2N‐ary choice tree model do predict that more attention to amounts should lead to the safe gamble being chosen (Noguchi & Stewart, 2014), but this pattern is not seen in Table 4. Decision by sampling does predict that more fixations to a gamble should increase the probability that it is chosen (Noguchi & Stewart, 2014), but because the model accumulates favourable comparisons, and because favourable comparisons are more likely for larger attribute values, decision by sampling incorrectly predicts an interaction between fixations to an attribute and its magnitude. Table 1 logs a tick for the first prediction and a cross for the second. The parallel constraint satisfaction model does not make clear predictions. However, if one assumes that activations of the nodes for gambles are associated with fixations to the attributes of those gambles, then the parallel constraint satisfaction model will predict the fixations‐to‐choice association. But as activation is higher when the difference in gamble values is larger, the parallel constraint satisfaction model incorrectly predicts a large interaction between fixations to an attribute and its magnitude. So, like decision by sampling, this is recorded in Table 1 with a tick and a cross. In summary, the simple model we sketch earlier is simpler than decision field theory, which assumes that attribute differences are accumulated, simpler than decision by sampling, which assumes that favourable comparisons are accumulated, and simpler than the parallel constraint satisfaction model, which assumes that probabilities and outcomes are integrated. (iv) For attribute values, about two thirds of the variance in choice predicted is unique to attribute values, and one third is shared with transitions. Similarly, for transitions, about two thirds of the variance in choice predicted is unique to transitions, and one third is shared with values. So, some of the effect of attribute values on choice is picked up in the eye movements, but some of the effect is not. Thus, whatever the model relating attribute values to choices, be it prospect theory or otherwise, only some of this processing is being picked up in eye movements. This would be true if eye movements were just a noisy measure of the process—but the second finding, that some of the eye movements predict choice but are unrelated to attribute values, rules this out as a complete account: Choices are affected by eye movements in a process that is independent of the attribute values. That is, at least some of the effect of eye movements on choice has nothing to do with the choice options people face. This places an upper bound on the performance of models relating attribute values to choices—a good deal of variance in choice comes from eye movements that are nothing to do with attribute values.

## Conclusion

We have analysed the eye movements made in simple risky decisions. Key results are listed in Table 1. In the first part of our analysis, we explored the relationship between attribute values and eye movements. People fixated the probabilities and amounts on the screen, revisiting them. They fixated the different probabilities and amounts about equally often, making more within‐gamble probability‐amount transitions. The pattern of fixations and transitions was the same across different choices—the eye movements did not depend on the probabilities and amounts on offer, except for finding that people made many more fixations (of the same types) before choosing when gambles were similar in probability. The pattern of eye movements was constant over time, except for a developing gaze bias, in which people fixated the attributes of the gamble they ultimately chose more often as they get closer to choosing.

In the second part of our analysis, we explored the relationship between eye movements and choice. The relationship is simpler than that predicted by existing models: People chose the gamble they most often fixated. The link between eye movements and choice overlaps only partially with the link between attribute values and choice. The implications are important. First, eye movements can tell us about only part of the link between attribute values and choice. Second, given that the eye‐movement‐to‐choice link is about the same size in terms of variability explained as the attribute‐value‐to‐choice link, eye movements are tapping an aspect of risky choice that, thus far, has been explored in only a handful of studies.

## Figures and Tables

**Figure A1:**
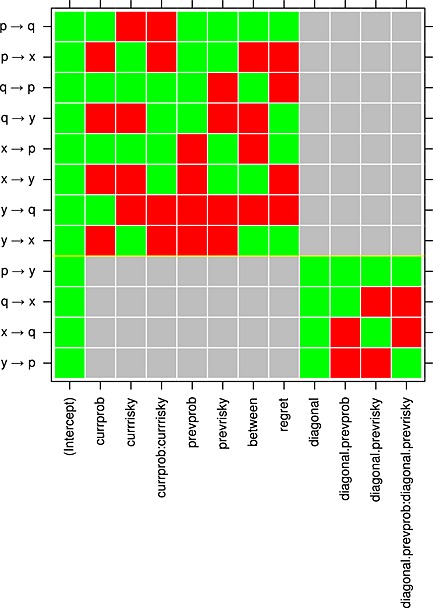
The design matrix for the saturated Poisson model of the transition counts. Each row corresponds to a cell in the transition count matrix show in Table 2. Each column refers to a coefficient from Table 6. Gray squares indicate 0 entries in the design matrix; red indicates –1 entries; green indicates +1 entries

## Appendix A

### The Poisson Regression Model of Transition Counts

Poisson regression was used to construct a saturated model of the transition counts in Table 2. In using this regression, our goal is to provide a statistical description of the counts, not to provide a psychological explanation for the counts. In the Poisson regression, each count in Table 2 is predicted by starting with an intercept and then multiplying the intercept by the coefficients that apply to that cell. In fact, Poisson regression is part of the generalized linear model and, as such, predicts the logarithm of the frequency of a cell as a linear sum of coefficients. To account for the within‐subjects nature of the design, we used mixed effects model, with by subjects intercepts and full by subjects slopes.

There are many ways to construct a saturated model of transition frequencies. All that is required is that 12 coefficients are used to predict the 12 non‐zero entries in Table 2. For example, we could set the intercept to the value of one cell and then use 11 coefficients to specify how to adjust that value to obtain the value in each of the other cells. This model would be saturated, perfectly fitting the data, but the coefficients would not have a ready interpretation. The trick is to choose a design matrix such that as many of the coefficients as possible have a simple interpretation.

Table A1 lists the terms we used in our Poisson regression. Figure A1 complements these verbal descriptions, showing exactly how each coefficient was used to predict the transition counts. Figure A1 is a design matrix, with each row in the matrix corresponding to a particular transition frequency from Table 2. Each column corresponds to a term from Table A1. Gray cells in Figure A1 indicate zeros, green cells indicate +1 entries, and red cells indicate –1 entries in the matrix. For example, the *currprob* term is described as indicating whether a transition ends on a probability in Table A1. The second column in Figure A1 shows that this is achieved by setting the *currprob* term to +1 for transitions ending on a probability and –1 for transitions ending on an amount. The design matrix (i.e., colouring in Figure A1) completely defines the terms (i.e., the descriptions in Table A1).

**Table A1:** Terms in the design matrix

Term		Description
	(Intercept)	The intercept is (the log of) the geometric mean of within and between transitions
*Within (p* ↔ *x and q* ↔ *y) and between (p* ↔ *q and x* ↔ *y) gamble transitions*		
	*currprob*	Transition is to a probability rather than an amount
	*currrisky*	Transition is to the risky option rather than the safe option
	*currprob*:*currrisky*	Interaction
	*prevprob*	Transition is from a probability rather than an amount
	*prevrisky*	Transition is from the risky option rather than the safe option
	*between*	Transition is between gambles rather than within. This is the *prevprob*:*currprob* interaction
	*regret*	Transition is from a good thing to a bad thing. This is the *prevrisky*:*currprob* interaction
*Diagonal transitions: Transitions that swap between both gambles and attributes (p* ↔ *y and q* ↔ *x)*		
	*diagonal*	Adjustment to the intercept if the transition is diagonal
	*diagonal*.*prevprob*	Transition is from a probability rather than amount
	*diagonal*.*prevrisky*	Transition is from the risky option rather than the safe option
	*diagonal*.*prevprob*:*diagonal*.*prevrisky*	Interaction

Consider, for example, the first eight rows of the design matrix labelled “*p* → *q*” through to “*y* → *x*.” These rows show how the *intercept* and the coefficients *currprob* through to *regret* are used to model the within‐gamble and between‐gamble transitions highlighted in Table 2. The first column in the design matrix indicates that modelling each cell frequency begins with the intercept. The intercept is the geometric mean of the frequency of within‐gamble and between‐gamble transitions. The next column *currprob* indicates whether the transition ends on a probability attribute (*p* or *q*) or an amount attribute (*x* or *y*). Green squares indicate that the *intercept* should be multiplied by the *currprob* coefficient, and red squares indicate that the *intercept* should be divided by the *currprob* coefficient. The *currrisky* column indicates whether the transition ends on an attribute of the risky gamble (*p* or *x*) or the safe gamble (*q* or *y*), and so on across the columns. Altogether, the *intercept* and the seven coefficients (eight parameters in total) are used to model the eight within‐gamble and between‐gamble transition frequencies.

The estimated coefficients are shown in column “exp(coef)” of Table 3. The coefficients are exponentially transformed. Because, in a Poisson regression, the transformed coefficients are multiplied to create cell frequencies, what matters is whether transformed coefficients are greater or less than 1. For each subject, the frequencies of each type of transition are given by multiplying the intercept by the coefficients. For example, the frequency of “*p* → *q*” transitions is given as follows: 1 × 49.04×.99/1.04/.97 × 1.11 × 1.01×.82 × 1.15, with the pattern of multiply, multiply, divide, divide, multiply, multiply, multiply, and multiply given by the +1, +1, –1, –1, +1, +1, +1, and +1 (green, green, red, red, green, green, green, and green) in the “*p* → *q*” row of the Figure A1 design matrix. Because the coefficients in Table 3 are the fixed effects from the mixed effect model, this is the estimate for the average participant.

In the main text, we describe the modelling of the within‐gamble between‐attribute transitions (i.e., *p* ↔ *x* and *q* ↔ *y*) and the between‐gamble within‐attribute transitions (i.e., *p* ↔ *q* and *x* ↔ *y*). These transitions comprise the majority, 89%, of the transitions. We have analysed the diagonal transitions carefully, but the results are not very interesting, and we do not report them here.

## References

[bdm1854-bib-0001] Arieli, A. , Ben‐Ami, Y. , & Rubinstein, A. (2011). Tracking decision makers under uncertainty. American Economic Journal: Microeconomics, 3, 68–76.

[bdm1854-bib-0002] Armel, K. C. , Beaumel, A. , & Rangel, A. (2008). Biasing simple choices by manipulating relative visual attention. Judgment and Decision Making, 3, 396–403.

[bdm1854-bib-0003] Bernoulli, D. (1954). Expositions of a new theory of the measurement of risk. Econometrica, 22, 23–36. (Original work published 1738)

[bdm1854-bib-0004] Birnbaum, M. H. (2004). Causes of Allais common consequence paradoxes: An experimental dissection. Journal of Mathematical Psychology, 48, 87–106.

[bdm1854-bib-0005] Birnbaum, M. H. (2008a). Evaluation of the priority heuristic as a descriptive model of risky decision making: Comment on Brandstätter, Gigerenzer, and Hertwig (2006). Psychological Review, 115, 253–260. 18211200 10.1037/0033-295X.115.1.253

[bdm1854-bib-0006] Birnbaum, M. H. (2008b). New paradoxes of risky decision making. Psychological Review, 115, 453–501. 10.1037/0033-295X.115.2.463 18426300

[bdm1854-bib-0007] Birnbaum, M. H. (2010). Testing lexicographic semiorders as models of decision making: Priority dominance, integration, interaction, and transitivity. Journal of Mathematical Psychology, 54, 363–386.

[bdm1854-bib-0008] Birnbaum, M. H. , & Chavez, A. (1997). Tests of theories of decision making: Violations of branch independence and distribution independence. Organizational Behavior and Human Decision Processes, 71, 161–194.

[bdm1854-bib-0009] Birnbaum, M. H. , & LaCroix, A. R. (2008). Dimension integration: Testing models without trade‐offs. Organizational Behavior and Human Decision Processes, 105, 122–133.

[bdm1854-bib-0010] Brandstätter, E. , Gigerenzer, G. , & Hertwig, R. (2006). The priority heuristic: Making choices without trade‐offs. Psychological Review, 113, 409–432. 16637767 10.1037/0033-295X.113.2.409 PMC2891015

[bdm1854-bib-0011] Busemeyer, J. R. , & Townsend, J. T. (1993). Decision field theory: A dynamic‐cognitive approach to decision making in an uncertain environment. Psychological Review, 100, 432–459. 8356185 10.1037/0033-295x.100.3.432

[bdm1854-bib-0012] Collins, A. M. , & Loftus, E. F. (1975). A spreading‐activation theory of semantic processing. Psychological Review, 82, 407–428. doi: 10.1037//0033-295X.82.6.407

[bdm1854-bib-0013] Dawes, R. M. (1979). The robust beauty of linear models of decision making. American Psychologist, 34, 571–582.

[bdm1854-bib-0014] Diederich, A. (1997). Dynamic stochastic models for decision making under time constraints. Journal of Mathematical Psychology, 41, 260–274. doi: 10.1006/jmps.1997.1167 9325121 10.1006/jmps.1997.1167

[bdm1854-bib-0015] Edwards, W. (1962). Subjective probabilities inferred from decisions. Psychological Review, 69, 109–135. 13889319 10.1037/h0038674

[bdm1854-bib-0016] Fiedler, S. , & Glöckner, A. (2012). The dynamics of decision making in risky choice: An eye‐tracking analysis. Frontiers in Psychology, 3, 335. doi: 10.3389/fpsyg.2012.00335 23162481 10.3389/fpsyg.2012.00335 PMC3498888

[bdm1854-bib-0017] Gigerenzer, G. , & Gaissmaier, W. (2011). Heuristic decision making. Annual Review of Psychology, 62, 451–482. doi: 10.1146/annurev-psych-120709-145346 10.1146/annurev-psych-120709-145346 21126183

[bdm1854-bib-0018] Glaholt, M. G. , & Reingold, E. M. (2009a). Stimulus exposure and gaze bias: A further test of the gaze cascade model. Attention, Perception, & Psychophysics, 71, 445–450. doi: 10.3758/APP.71.3.445 10.3758/APP.71.3.445 19304635

[bdm1854-bib-0019] Glaholt, M. G. , & Reingold, E. M. (2009b). The time course of gaze bias in visual decision tasks. Visual Cognition, 17, 1228–1243.

[bdm1854-bib-0020] Glaholt, M. G. , & Reingold, E. M. (2011). Eye movement monitoring as a process tracing methodology in decision making research. Journal of Neuroscience, Psychology, and Economics, 4, 125–146.

[bdm1854-bib-0021] Glöckner, A. , & Betsch, T. (2008a). Do people make decisions under risk based on ignorance? an empirical test of the priority heuristic against cumulative prospect theory. Organizational Behavior and Human Decision Processes, 107, 75–95. doi: 10.1016/j.obhdp.2008.02.003

[bdm1854-bib-0022] Glöckner, A. , & Betsch, T. (2008b). Modeling option and strategy choices with connectionist networks: Towards an integrative model of automatic and deliberate decision making. Judgment and Decision Making, 3, 215–228.

[bdm1854-bib-0023] Glöckner, A. , Fiedler, S. , Hochman, G. , Ayal, S. , & Hilbig, B. E. (2012). Processing differences between descriptions and experience: A comparative analysis using eye‐tracking and physiological measures. Frontiers in Cognitive Science, 3, 173. 10.3389/fpsyg.2012.00173 PMC3374477 22707943

[bdm1854-bib-0024] Glöckner, A. , Heinen, T. , Johnson, J. G. , & Raab, M. (2012). Network approaches for expert decisions in sports. Human Movement Science, 31, 318–333. doi: 10.1016/j.humov.2010.11.002 21798611 10.1016/j.humov.2010.11.002

[bdm1854-bib-0025] Glöckner, A. , & Herbold, A.‐K. (2011). An eye‐tracking in risky decisions: Evidence for compensatory strategies based on automatic processes. Journal of Behavioral Decision Making, 24, 71–98. doi: 10.1002/bdm.684

[bdm1854-bib-0026] Gold, J. I. , & Shadlen, M. N. (2007). The neural basis of decision making. Annual Review of Neuroscience, 30, 535–574. 10.1146/annurev.neuro.29.051605.113038 17600525

[bdm1854-bib-0027] Horstmann, N. , Ahlgrimm, A. , & Glöckner, A. (2009). How distinct are intuition and deliberation? An eye‐tracking analysis of instruction‐induced decision modes. Judgment and Decision Making, 4, 335–354. Retrieved from http://journal.sjdm.org/9323/jdm9323.pdf

[bdm1854-bib-0028] Huang, K. , Sen, S. , & Szidarovszky, F. (2012). Connections among decision field theory models of cognition. Journal of Mathematical Psychology, 56, 287–296. doi: 10.1016/j.jmp.2012.07.005

[bdm1854-bib-0029] Janowski, V. , & Rangel, A. (2012). Variation in loss aversion is associated with differential attention to losses. Manuscript submitted for publication.

[bdm1854-bib-0030] Johnson, E. J. , Schulte‐Mecklenbeck, M. , & Willemsen, M. C. (2008). Process models deserve process data: Comment on Brandstätter, Gigerenzer, and Hertwig (2006). Psychological Review, 115, 263–273. 18211202 10.1037/0033-295X.115.1.263

[bdm1854-bib-0031] Kahneman, D. , & Tversky, A. (1979). Prospect theory: An analysis of decision under risk. Econometrica, 47, 263–291.

[bdm1854-bib-0032] Krajbich, I. , Armel, C. , & Rangel, A. (2010). Visual fixations and the computation and comparison of value in simple choice. Nature Neuroscience, 13, 1292–1298. doi: 10.1038/nn.2635 20835253 10.1038/nn.2635

[bdm1854-bib-0033] Krajbich, I. , & Rangel, A. (2011). Multialternative drift‐diffusion model predicts the relationship between visual fixations and choice in value‐based decisions. Proceedings of the National Academy of Sciences of the United States of America, 108, 13852–13857. doi: 10.1073/pnas.1101328108 21808009 10.1073/pnas.1101328108 PMC3158210

[bdm1854-bib-0034] Laming, D. R. J. (1968). Information theory of choice‐reaction times. London: Academic.

[bdm1854-bib-0035] Liversedge, S. P. , & Findlay, J. M. (2000). Saccadic eye movements and cognition. Trends in Cognitive Sciences, 4, 6–14. doi: 10.1016/S1364-6613(99)01418-7 10637617 10.1016/s1364-6613(99)01418-7

[bdm1854-bib-0036] Loomes, G. , Navarro‐Martinez, D. , Isoni, A. , & Butler, D. J. (2012). A sequential sampling model of choice. Unpublished manuscript.

[bdm1854-bib-0037] Loomes, G. , & Sugden, R. (1982). Regret theory: An alternative theory of rational choice under uncertainty. Economic Journal, 92, 805–824.

[bdm1854-bib-0038] Mullett, T. L. , & Stewart, N. (2014). Implications of visual attention phenomena for models of preferential choice. Manuscript submitted for publication. 10.1037/dec0000049 PMC5058407 27774490

[bdm1854-bib-0039] Noguchi, T. , & Stewart, N. (2014). In the attraction, compromise, and similarity effects, alternatives are repeatedly compared in pairs on single dimensions. Cognition, 132, 44–56. doi: 10.1016/j.cognition.2014.03.006 24762922 10.1016/j.cognition.2014.03.006

[bdm1854-bib-0040] Orquin, J. L. , & Mueller Loose, S. (2013). Attention and choice: A review of decision making and eye tracking studies. Acta Psychologica, 144, 190–206. doi: 10.1016/j.actpsy.2013.06.003 23845447 10.1016/j.actpsy.2013.06.003

[bdm1854-bib-0041] Pachur, T. , Hertwig, R. , Gigerenzer, G. , & Brandstätter, E. (2013). Testing process predictions of models of risky choice: A quantitative model comparison approach. Frontiers in Psychology, 4. doi: 10.3389/fpsyg.2013.00646 10.3389/fpsyg.2013.00646 PMC3784771 24151472

[bdm1854-bib-0042] Payne, J. W. , Bettman, J. R. , & Johnson, E. J. (1988). Adaptive strategy selection in decision‐making. Journal of Experimental Psychology: Learning, Memory, and Cognition, 14, 534–552. doi: 10.1037//0278-7393.14.3.534

[bdm1854-bib-0043] Payne, J. W. , & Braunstein, M. L. (1978). Risky choice: Examination of information acquisition behavior. Memory & Cognition, 6, 554–561. doi: 10.3758/BF03198244 24203389 10.3758/BF03198244

[bdm1854-bib-0044] Quiggin, J. (1993). Generalized expected utility theory: The rank‐dependent model. Norwell, MA: Kluwer Academic Publishers.

[bdm1854-bib-0045] Ratcliff, R. , & Rouder, J. N. (1998). Modeling response times for two‐choice decisions. Psychological Science, 9, 347–356. doi: 10.1111/1467-9280.00067

[bdm1854-bib-0046] Rayner, K. (1998). Eye movements in reading and information processing: 20 years of research. Psychological Bulletin, 124, 372–422. doi: 10.1037/0033-2909.124.3.372 9849112 10.1037/0033-2909.124.3.372

[bdm1854-bib-0047] Rayner, K. (2009). Eye movements and attention in reading, scene perception, and visual search. Quarterly Journal of Experimental Psychology, 62, 1457–1506. doi: 10.1080/17470210902816461 10.1080/17470210902816461 19449261

[bdm1854-bib-0048] Rayner, K. , Pollatsek, A. , Ashby, J. , & Clifton Jr, C. (2012). The psychology of reading (2nd ed). New York: Psychology Press.

[bdm1854-bib-0049] Reutskaja, E. , Nagel, R. , Camerer, C. F. , & Rangel, A. (2011). Search dynamics in consumer choice under time pressure: An eye‐tracking study. American Economic Review, 101, 900–926.

[bdm1854-bib-0050] Roe, R. M. , Busemeyer, J. R. , & Townsend, J. T. (2001). Multialternative decision field theory: A dynamic connectionist model of decision making. Psychological Review, 108, 370–392. 11381834 10.1037/0033-295x.108.2.370

[bdm1854-bib-0051] Rosen, L. D. , & Rosenkoetter, P. (1976). Eye fixation analysis of choice and judgment with multiattribute stimuli. Memory & Cognition, 4, 747–752. doi: 10.3758/BF03213243 21287006 10.3758/BF03213243

[bdm1854-bib-0052] Russo, J. E. (2011). Eye fixations as a process trace In Schulte‐Mecklenbeck M., Kühberger A., & Ranyard R. (Eds.), A handbook of process tracing methods for decision research: A critical review and user's guide (pp. 43–64). New York: Psychology Press.

[bdm1854-bib-0053] Russo, J. E. , & Dosher, B. A. (1983). Strategies for multiattribute binary choice. Journal of Experimental Psychology: Learning, Memory, and Cognition, 9, 676–696. doi: 10.1037//0278-7393.9.4.676 10.1037//0278-7393.9.4.676 6227682

[bdm1854-bib-0054] Russo, J. E. , & Leclerc, F. (1994). An eye‐fixation analysis of choice processes for consumer nondurables. Journal of Consumer Research, 21, 274–290.

[bdm1854-bib-0055] Savage, L. J. (1954). The foundations of statistics. New York: Wiley.

[bdm1854-bib-0056] Schotter, E. R. , Berry, R. W. , McKenzie, C. R. M. , & Rayner, K. (2010). Gaze bias: Selective encoding and liking effects. Visual Cognition, 18, 1113–1132. doi: 10.1080/13506281003668900

[bdm1854-bib-0057] Schotter, E. R. , Gerety, C. , & Rayner, K. (2012). Heuristics and criterion setting during selective encoding in visual decision making: Evidence from eye movements. Visual Cognition, 20, 1110–1129. doi: 10.1080/13506285.2012.735719 23264730 10.1080/13506285.2012.735719 PMC3524580

[bdm1854-bib-0058] Shi, S. W. , Wedel, M. , & Pieters, F. G. M. R. (2013). Information acquisition during online decision making: A model‐based exploration using eye‐tracking data. Management Science, 59, 1009–1026. doi: 10.1287/mnsc.1120.1625

[bdm1854-bib-0059] Shimojo, S. , Simion, C. , Shimojo, E. , & Scheier, C. (2003). Gaze bias both reflects and influences preference. Nature Neuroscience, 6, 1317–1322. doi: 10.1038/nn1150 14608360 10.1038/nn1150

[bdm1854-bib-0060] Simion, C. , & Shimojo, S. (2006). Early interactions between orienting, visual sampling and decision making in facial preference. Vision Research, 46, 3331–3335. doi: 10.1016/j.visres.2006.04.019 16765404 10.1016/j.visres.2006.04.019

[bdm1854-bib-0061] Simion, C. , & Shimojo, S. (2007). Interrupting the cascade: Orienting contributes to decision making even in the absence of visual stimulation. Perception & Psychophysics, 69, 591–595. doi: 10.3758/BF03193916 17727112 10.3758/bf03193916

[bdm1854-bib-0062] Stewart, N. (2009). Decision by sampling: The role of the decision environment in risky choice. Quarterly Journal of Experimental Psychology, 62, 1041–1062. doi: 10.1080/17470210902747112 10.1080/17470210902747112 19306161

[bdm1854-bib-0063] Stewart, N. , Chater, N. , & Brown, G. D. A. (2006). Decision by sampling. Cognitive Psychology, 53, 1–26. doi: 10.1016/j.cogpsych.2005.10.003 16438947 10.1016/j.cogpsych.2005.10.003

[bdm1854-bib-0064] Stewart, N. , Reimers, S. , & Harris, A. J. L. (in press). On the origin of utility, weighting, and discounting functions: How they get their shapes and how to change their shapes. Management Science. doi: 10.1287/mnsc.2013.1853

[bdm1854-bib-0065] Stewart, N. , & Simpson, K. (2008). A decision‐by‐sampling account of decision under risk In Chater N., & Oaksford M. (Eds.), The probabilistic mind: Prospects for Bayesian cognitive science (pp. 261–276). Oxford, England: Oxford University Press.

[bdm1854-bib-0066] Su, Y. , Rao, L. L. , Sun, H. Y. , Du, X. L. , Li, X. S. , & Li, S. (2013). Is making a risky choice based on a weighting and adding process? An eye‐tracking investigation. Journal of Experimental Psychology: Learning, Memory, and Cognition, 39, 1765–1780. doi: 10.1037/a0032861 10.1037/a0032861 23687917

[bdm1854-bib-0067] Teodorescu, A. R. , & Usher, M. (2013). Disentangling decision models: From independence to competition. Psychological Review, 120, 1–38. doi: 10.1037/a0030776 23356779 10.1037/a0030776

[bdm1854-bib-0068] Tversky, A. , & Kahneman, D. (1992). Advances in prospect theory: Cumulative representation of uncertainty. Journal of Risk and Uncertainty, 5, 297–323.

[bdm1854-bib-0069] Usher, M. , & McClelland, J. L. (2001). The time course of perceptual choice: The leaky, competing accumulator model. Psychological Review, 108, 550–592. 11488378 10.1037/0033-295x.108.3.550

[bdm1854-bib-0070] Vickers, D. (1970). Evidence for an accumulator model of psychophysical discrimination. Ergonomics, 13, 37–58. 5416868 10.1080/00140137008931117

[bdm1854-bib-0071] von Neumann, M. , & Morgenstern, O. (1947). Theory of games and economic behavior (2nd ed). Princeton, NJ: Princeton University Press.

[bdm1854-bib-0072] Wollschläger, L. M. , & Diederich, A. (2012). The 2N‐ary choice tree model for N‐alternative preferential choice. Frontiers in Psychology, 3, 1–11. doi: 10.3389/fpsyg.2012.00189 22723788 10.3389/fpsyg.2012.00189 PMC3378970

[bdm1854-bib-0073] Yarbus, A. L. (1967). Eye movements and vision. New York: Plenum Press.

